# Transcutaneous cervical vagus nerve stimulation improved motor cortex excitability in healthy adults: a randomized, single-blind, self-crossover design study

**DOI:** 10.3389/fnins.2023.1234033

**Published:** 2023-10-03

**Authors:** Meng-Xin Wang, Aihaiti Wumiti, Yao-Wen Zhang, Xue-Sheng Gao, Zi Huang, Meng-Fei Zhang, Zhi-Yong Peng, Yoshitaka Oku, Zhi-Ming Tang

**Affiliations:** ^1^Department of Rehabilitation Medicine, Yuedong Hospital, The Third Affiliated Hospital of Sun Yat-sen University, Meizhou, China; ^2^Department of Rehabilitation Medicine, The Third Affiliated Hospital of Sun Yat-sen University, Guangzhou, China; ^3^Rehabilitation Medicine Department, Affiliated Hospital of Weifang Medical University, Weifang, China; ^4^Department of Physiology, Hyogo Medical University, Hyogo, Japan

**Keywords:** noninvasive vagus nerve stimulation, motor cortex excitability, neuromodulation, transcranial magnetic stimulation, motor evoke potential

## Abstract

**Purpose:**

To investigate the effect of transcutaneous cervical vagus nerve stimulation (tcVNS) on motor cortex excitability in healthy adults.

**Method:**

Twenty eight healthy subjects were assigned to receive real and sham tcVNS for 30 min. The interval between the real and sham conditions was more than 24 h, and the sequence was random. The central and peripheral motor-evoked potential (MEP) of the right first dorsal interosseous (FDI) muscle was measured by transcranial magnetic stimulation (TMS) before and after stimulation. MEP latency, MEP amplitude and rest motor threshold (rMT) were analyzed before and after stimulation.

**Results:**

MEP amplitude, MEP latency and rMT had significant interaction effect between time points and conditions (*p* < 0.05). After real stimulation, the MEP amplitude was significantly increased (*p* < 0.001). MEP latency (*p* < 0.001) and rMT (*p* = 0.006) was decreased than that of baseline. The MEP amplitude on real condition was higher than that of sham stimulation after stimulation (*p* = 0.027). The latency after the real stimulation was significantly shorter than that after sham stimulation (*p* = 0.005). No significantly difference was found in rMT after stimulation between real and sham conditions (*p* > 0.05).

**Conclusion:**

tcVNS could improve motor cortex excitability in healthy adults.

## 1. Introduction

Stroke, brain trauma, and other neurological diseases often result in extensive and lasting motor dysfunction (Nazarova et al., 2021). In some cases, such as when upper limb function is affected, the recovery process is long, which seriously affects the life of patients and places a heavy burden on the family and society (Zhao and Huang, 2019). Rehabilitation training is an important approach to improve patients’ motor function, but optimal solutions have not yet been found (Gittler and Davis, 2018).

Vagus nerve stimulation (VNS), one of many explorations of motor dysfunction rehabilitation (Kimberley et al., 2018; Li et al., 2022), can be divided into invasive and non-invasive VNS (nVNS) (Ramos-Castaneda et al., 2022). Previous studies have reported its application in cases of epilepsy (Ryvlin et al., 2021; Oehrn et al., 2022), depression (Conroy and Holtzheimer, 2021; Evensen et al., 2022), and tinnitus (De Ridder et al., 2021). Few studies have investigated its effect on motor function (Dawson and Abdul-Rahim, 2022). Porter et al. attempted to explore the effect of VNS on the motor cortex and found that repeated pairings between VNS and compression or rotation task training increased the motor cortex area of the distal and proximal forelimbs of rats, respectively (Porter et al., 2012). Dawson et al. reported that after invasive VNS (iVNS) was performed by wrapping an electrode around the left cervical vagus nerve of stroke patients, the upper limb function improved significantly better than in a regular rehabilitation training group or sham stimulation group (Dawson et al., 2021), indicating that VNS combined with motor training can improve the motor function of upper limbs. However, as this method is invasive and has high technical requirements, it is unlikely to be widely used.

nVNS includes tcVNS and transcutaneous auricular VNS (taVNS) (Yang et al., 2018; Farmer et al., 2020; Li et al., 2022; van Midden et al., 2023). Most researches on taVNS come from the study on auricular points in traditional Chinese medicine (Wang et al., 2022; Zhang et al., 2022). tcVNS is also an important kind of vagus nerve stimulation (Frangos and Komisaruk, 2017), especially with potential advantages in parameter standardization (Gurel et al., 2020). After exiting the cranial cavity through the jugular foramen, the bilateral vagus nerves run together with the common carotid artery and internal jugular vein within the carotid sheath. Their location near the surface can be identified by the medial edge of the bilateral sternocleidomastoid muscle, making it easy to locate and place stimulation electrodes. Also, the tcVNS stimulate the same branch of vagus nerve of iVNS (Gurel et al., 2020). Several pilot studies have shown that nVNS might improve motor function after stroke better than separate rehabilitation training (Capone et al., 2017; Yang et al., 2018). Compared with iVNS, which is not only expensive but carries high surgical risk, tcVNS is safer, more comfortable, and easier to carry out (Yuan and Silberstein, 2016; Redgrave et al., 2018). Moreover, it greatly reduces medical cost, which is of great clinical significance. However, although a large number of studies have been conducted on tcVNS in recent decades, the mechanism through which it improves motor function remains unclear. Objective evaluation indicators of neurophysiological effects under tcVNS are also lacking. The explanations given by most existing hypotheses rest on synaptic plasticity in neurotransmitter promotion-related pathways, and few studies have focused on the effects of VNS on motor cortex excitability.

TMS is a widely used noninvasive method of measuring motor cortex excitability (Tang et al., 2019; Zhang et al., 2022). MEP can be obtained in the dominant muscles by delivering a single pulse of magnetic stimulation in the motor cortex. MEP amplitude and MEP latency can be used to evaluate corticospinal excitability (Udupa and Chen, 2013). The increase of MEP amplitude and the shortening of cortical latency indicate increased cortical excitability.

In this study, we delivered an interference current to the neck skin to stimulate the vagus nerves’ bilateral cervical branches and assessed its safety in healthy subjects. The amplitude and latency of MEP were observed to explore the effect of bilateral tcVNS on motor cortex excitability.

## 2. Method

### 2.1. Subject

According to the preliminary experiment data, the study involved 28 healthy volunteers between the ages of 20 and 38 years old with an average age of 24.8 ± 4.4. Nine were male, and 19 were female. All participants met the inclusion criteria of being ① aged 20–35 and ② right-handed and exclusion criteria of having ① a history of mental disorder or nervous system disease, ② pacemakers, cochlear implants, dental implants, or other metal implants, and ③ severe cervical spondylosis, including cervical instability. Participants were asked to avoid alcohol, tea, and coffee, which might affect brain activity, and to keep a relatively regular routine during the study. All participants signed written informed consent to participate in the study. The study protocol was approved by the Ethics Committee of Yuedong Hospital, the Third Affiliated Hospital of Sun Yat-Sen University (approval document number: 2021–7), and the Chinese clinical trial registration number is ChiCTR2100054543.

### 2.2. Study design

The study used a self-crossover, randomized, single-blind controlled study design. All subjects received tcVNS in real and sham stimulation conditions on two different days with an interval of more than 24 h. The real and sham stimulus sequences were randomly assigned based on computer-generated numbers, which were kept by one person. The experiment was carried out by two operators. One person performed the tcVNS stimulation. The other operator, who did not know the stimulation conditions, used TMS to measure MEP (latency and amplitude) before and after the real or sham condition.

### 2.3. tcVNS stimulation

The experiment was conducted in a quiet and comfortable environment. The subjects sat in a comfortable position in a chair with arms on either side and were asked to remain relaxed.

**Figure 1:**
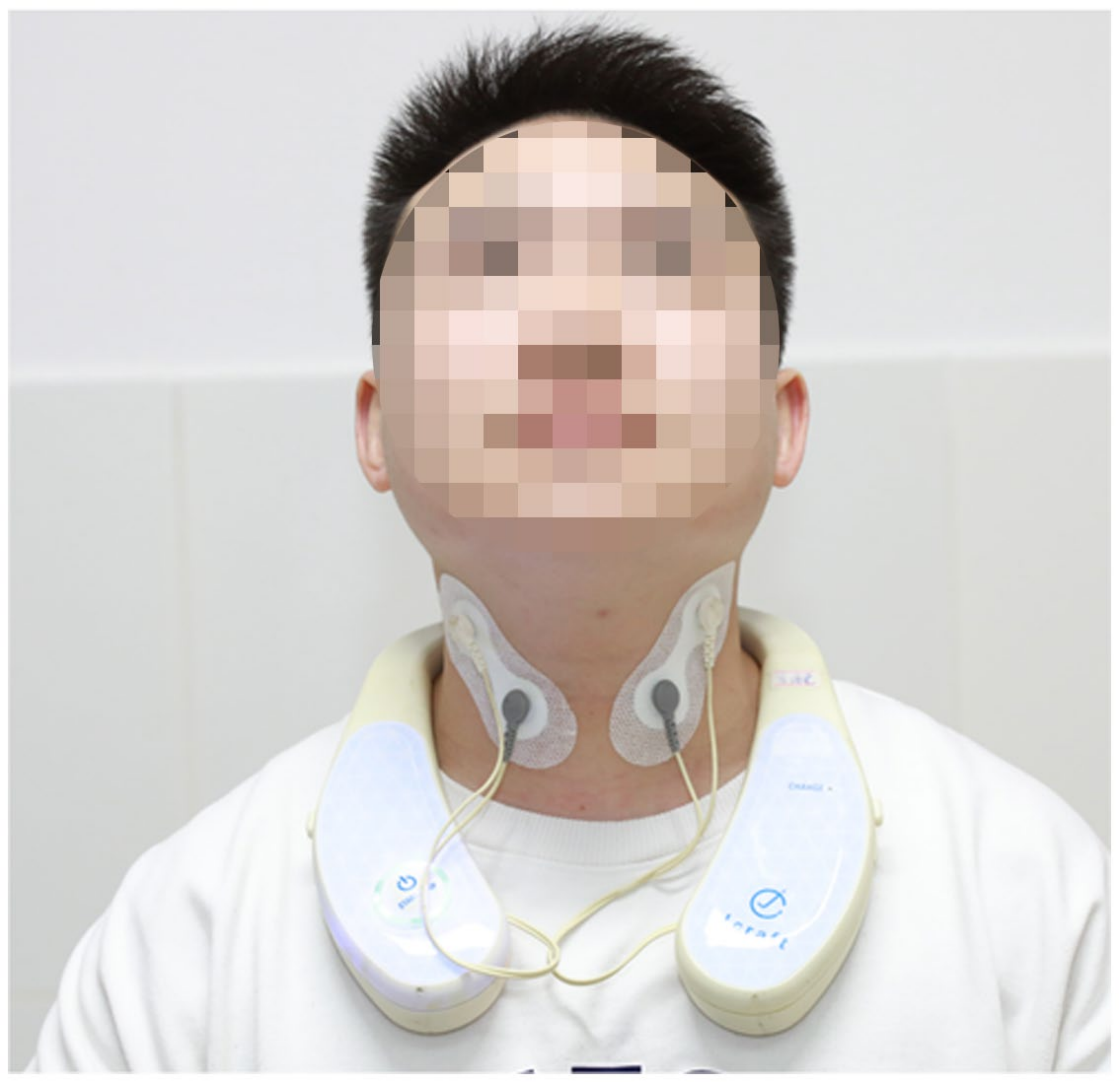
The stimulation electrode’s location of tcVNS.

The application conditions of tcVNS were as follows (Nagami et al., 2019): The interference current stimulation equipment was made in Japan (GentleAce neckgear J1000; J Craft, Osaka, Japan). The two pairs of electrodes (gray and white; see Figure 1) were 4 cm apart, delivering an alternating current of different frequencies (2000 Hz and 2050 Hz, respectively) to generate an interference current with a phase difference frequency of 50 Hz. The stimulation position was the bilateral cervical branch of the vagus nerve. The subject was instructed to sit in a chair, and the researchers cleaned the subject’s skin on the neck with a 75% medical alcohol swab. Two pairs of electrodes, each consisting of one gray and one white electrode, were affixed onto separate skin patches with a 4 cm distance between them. The patches were symmetrically placed bilaterally on the neck. The gray electrodes were positioned at the intersection of the thyroid cartilage and the front edge of the sternocleidomastoid muscle. The white electrodes were placed in a straight line between the mandibular angle and the corresponding gray electrodes on both sides (as depicted in Figure 1). Stimulation intensity was adjusted using a graded approach control software on a tablet computer, with a minimum stimulation intensity of 0.1 mA. However, each subsequent level of intensity did not necessarily result in an equivalent increase in actual output current. Under the real stimulation condition, to ensure safety and avoid excessive stimulation, the experimenters adjusted the stimulation intensity to maintain it between a range of 2.5 mA to 3.0 mA for a duration of 30 min. As individuals have different sensory thresholds, the sensations experienced by participants during the stimulation were not uniform. However, most participants reported tolerable vibratory sensations. This precise approach to controlling stimulation intensity ensured the safety of the participants. Under the sham stimulation condition, the setting was maintained for a total of 30 min with the device turn off To maintain single blinding, a designated data analyst conducted the statistical analysis without knowledge of the stimulation conditions and remained unaware of whether the data came from the real or sham stimulation conditions until all analyses were completed. This stringent approach minimized the risk of biased data analysis and enhanced the credibility and robustness of the study findings.

### 2.4. Transcranial magnetic stimulation

**Figure 2:**
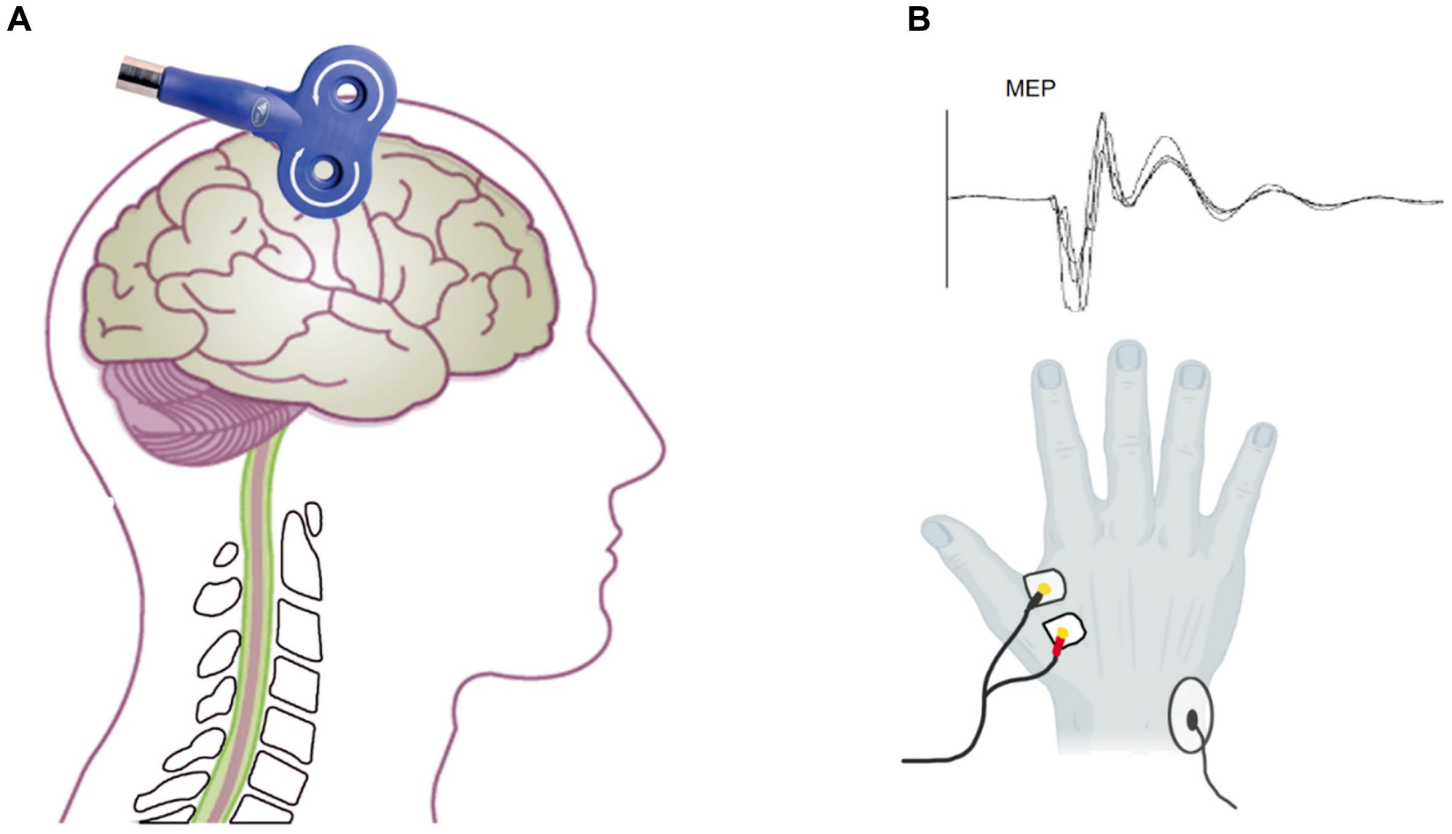
Assessment of MEP by transcranial magnetic stimulation. **(A)** The coil located on the M1 to assess the MEP amplitude and latency. **(B)** The electrode on the FDI (below) and the MEP waveform.

The method was as explained in our previous studies (Tang et al., 2019; Zhang J. et al., 2022; Zhang M. F. et al., 2022). The subject sat relaxed in a high-backed chair with armrests which supported their forearms, back, and legs. The subject was advised not to move their body during the rest motor threshold (rMT) and MEP measurements to keep the coil in the same position (Figure 2).

A facial scrub was used to clean the skin of the subject’s first dorsal interosseous (FDI) muscle to ensure a low skin impedance of ≤10 kΩ. A pair of 1 cm diameter bipolar disposable surface electrodes was attached to the FDI muscle of the right hand at a distance of 1.5 cm (Figure 2C). The ground electrode was placed on the right ulnar styloid process. The MEP was recorded by EMG (Neuron-Spectrum-5, Russia) with filtering set at 5–10KHz, sampling rate at 25KHz, and scanning speed at 5 ms/div.

The subjects kept their bilateral upper limb muscles relaxed and wore positioning caps. C3 was used as the reference point to locate the hand representative region in the primary motor cortex according to the international 10–20 EEG localization standard. TMS equipment (Wuhan Yiruide Medical Equipment New Technology Co., Ltd., China) was used with a figure-eight coil, which consists of two small circular coils with a diameter of 9 cm and opposite current directions overlapping each other, to measure MEP. The coil was tangent to the scalp, and the handle was at a 45-degree angle from the midline of the body (Figure 2A). The 70% maximum machine intensity of the TMS was selected as the baseline to initiate stimulation. The location around C3 that consistently elicited large-amplitude MEP was identified as the “hotspot.” According to previous guidelines, we employed the relative frequency method to determine the TMS output intensity (Rossini et al., 2015). It was reduced as it approached the threshold until the lowest output intensity was able to evoke at least 5 MEPs with amplitudes >50 μV of 10 consecutive TMS stimulations. This output intensity was defined as rMT. Before and after tcVNS stimulation, we stimulated the “hotspot” with 120% rMT intensity TMS to obtain the corticospinal MEP (Tang et al., 2019; Zhang et al., 2022). Any MEPs that were contaminated by activity in other muscles or had an amplitude less than 50 μV were excluded from the analysis. Ten MEP were recorded (Yang et al., 2017; Sun et al., 2019; Tang et al., 2019), and their peak MEP amplitude and latency were calculated (Figure 2C).The mean of the ten MEPs was used for further analysis.

### 2.5. Safety monitoring

The subject’s heart rate and blood oxygen were monitored during the study by a pulse oximetry device (POD3, Heal Force, Ltd. Shenzhen, China) worn on their right index finger. The experiment was stopped immediately if the heart rate fell below 60 or adverse reactions occurred, such as difficult breathing, unbearable skin tingling, or dizziness.

### 2.6. Statistical analysis

The G Power 3.1 statistical tool was used to determine the required sample size for the present trials based on the results of pre-experiments and the desired statistical power of 80% with statistical significance at *p* < 0.05 (2-tailed test). The analysis showed that at least 20 subjects were required for the study.

SPSS 23.0 software was used to analyze the data. We used the Shapiro–Wilk test to perform normality distribution. Data were expressed as mean ± standard deviation (SD); two factor (time * condition) repeated measurement ANOVA was used to compare the differences among each assessment point and post-Hoc using Bonferroni correction was used to compare the differences in MEP amplitude, MEP latency and rMT before and after stimulation within each condition. *p* < 0.05 was considered statistically significant.

## 3. Results

### 3.1. Changes of MEP amplitude

**Figure 3:**
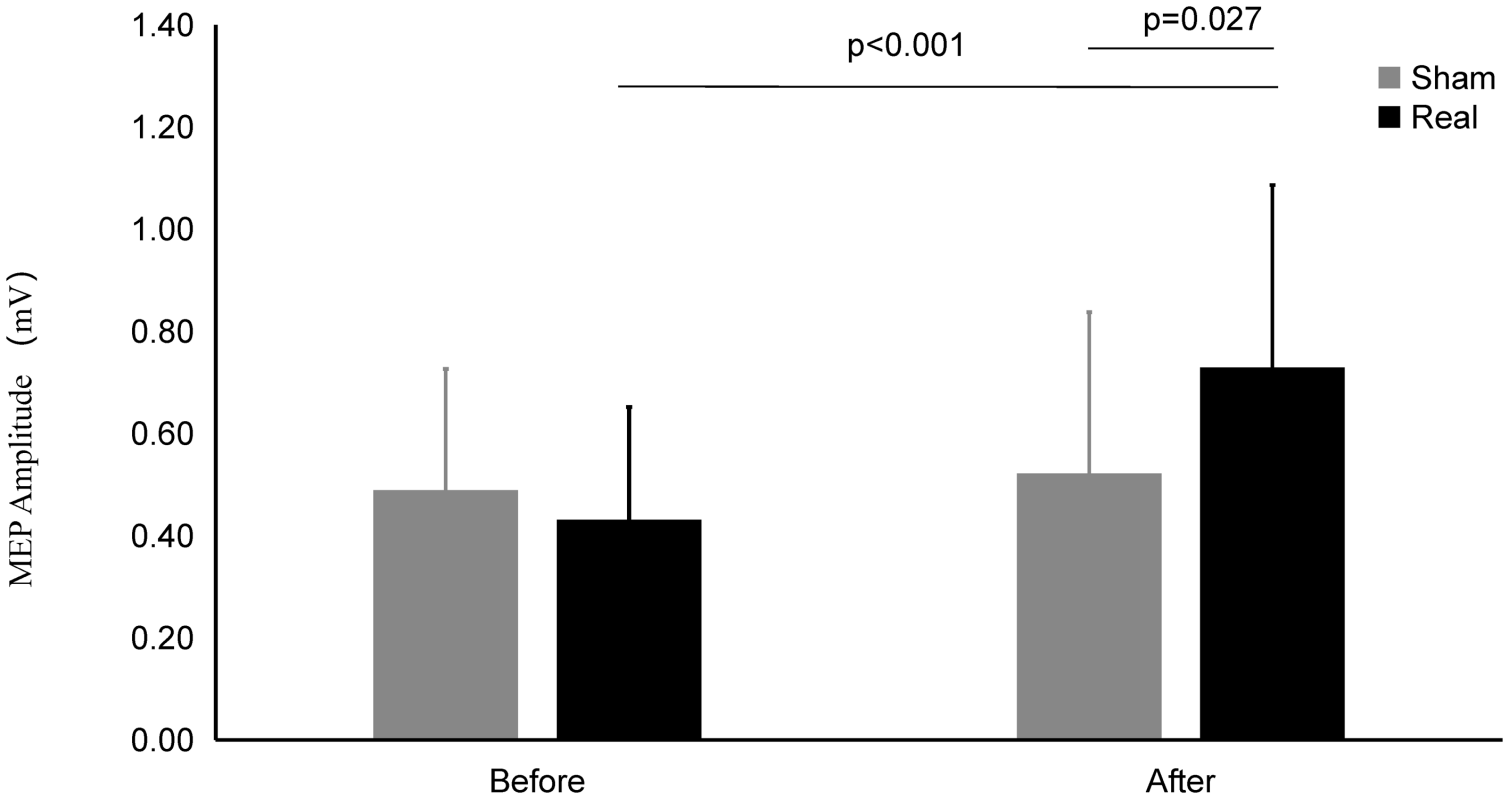
MEP amplitude on Real and Sham condition. The MEP Amplitude was significantly increased on real condition after tcVNS (*p* < 0.001). After tcVNS, MEP Amplitude was higher in real condition compared to the sham condition (*p* = 0.027).

There was a significant interaction effect between measurement time points and conditions (*F*_1, 27_ = 7.039, *p* = 0.013) (Figure 3). Significantly main effect was found in time points (*F*_1,27_ = 29.473, *p* < 0.001). No significant main effect was found in conditions (*F*_1,27_ = 2.307, *p* = 0.140).

After real stimulation, the MEP amplitude (0.73 ± 0.36 mV) was significantly higher than that before stimulation (0.43 ± 0.22 mV) (*t* = 4.185, *p* < 0.001). However, the MEP amplitude after the sham stimulation (0.52 ± 0.31 mV) did not change significantly compared with that before the stimulation (0.49 ± 0.24 mV) (*t* = 0.784, *p* = 0.440). There was significant difference of MEP amplitude between the real and sham condition after stimulation (*t* = 2.342, *p* = 0.027).

### 3.2. Change of the MEP latency

**Figure 4:**
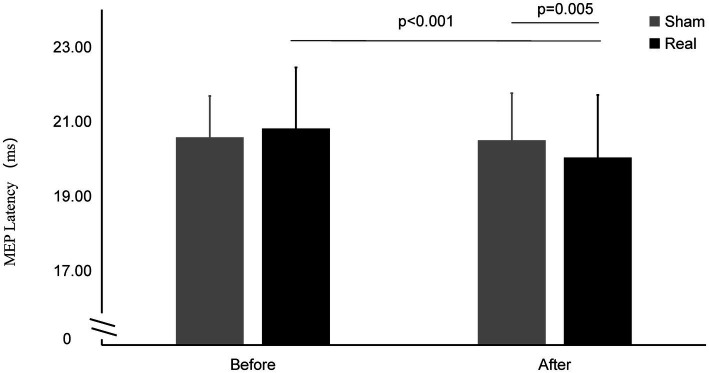
MEP latency on Real and Sham condition. MEP latency significantly decreased on real condition after tcVNS (*p* < 0.001). After tcVNS, MEP latency was shorten real condition compared to the sham condition (*p* = 0.005).

As shown in Figure 4, there was a significant interaction effect between time points and conditions (*F*_1,27_ = 21.316, *p* < 0.001). Significantly main effect was found in time points (*F*_1,27_ = 19.746, *p* < 0.001). No significant main effect was found in conditions (*F*_1,27_ = 0.621, *p* = 0.438).

The MEP latency after real stimulation (20.03 ± 1.68 ms) was significantly shorter than before stimulation (20.81 ± 1.64 ms) (*t* = 6.173, *p* < 0.001), while there was no statistically significant change in the MEP latency before and after the sham stimulation (20.57 ± 1.11 ms vs. 20.50 ± 1.26 ms) (*t* = 0.649, *p* = 0.522). The latency after the real stimulation was significantly shorter than that after sham stimulation (t = 3.093, *p* = 0.005).

### 3.3. Change of the rMT

**Figure 5:**
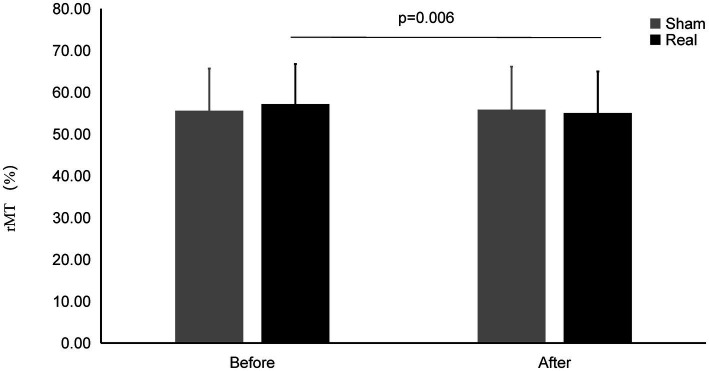
rMT on Real and Sham condition. rMT significantly decreased on real condition after tcVNS (*p* = 0.006).

As shown in Figure 5, there was a significant interaction effect between measurement time points (*F*_1,27_ = 9.097, *p* = 0.006). No significant main effect was found in time points (*F*_1,27_ = 3.645, *p* = 0.067). No significant main effect was found in conditions (*F*_1,27_ = 0.248, *p* = 0.622).

The rMT after real stimulation (55.04 ± 9.95%) was significantly decreased than before stimulation (57.18 ± 9.59%) (*t* = 2.993, *p* = 0.006), while there was also no statistically significant change in the rMT before and after the sham stimulation (55.58 ± 10.10% vs. 55.83 ± 10.32%) (*t* = 0.461, *p* = 0.648). There was no significant difference after the real stimulation and that of after sham stimulation (*t* = 0.866, *p* = 0.394).

### 3.4. Safety

At the onset of real stimulation, three participants experienced mild skin tingling, which was tolerable and quickly adapted to. No other adverse reactions were reported.

## 4. Discussion

tcVNS can now safely stimulate the vagus nerve without injury, and it is becoming widely used in various functional disorders in stroke rehabilitation. However, how tcVNS activates the relevant brain regions and produces central effects to improve motor function remains to be explored. This study investigated the effect of tcVNS on the excitability of the motor cortex. The increase in MEP amplitude and the shortening of latency prompted the corticospinal tract, suggesting that tcVNS could significantly improve the excitability of the motor cortex.

nVNS includes both tcVNS and taVNS. Some previous studies have investigated the taVNS on the motor function, but few have investigated this in relation to tcVNS (Frangos and Komisaruk, 2017). Redgrave et al. combined taVNS with post-stroke upper limb rehabilitation, and found improvement in motor function in a pilot study (Redgrave et al., 2018). Capone et al. reported that taVNS combined with robot-assisted rehabilitation might promote the motor function of the upper limb (Capone et al., 2017). However, they only assessed the clinical effectiveness and did not investigate the mechanism. Our previous studies have found that stimulation of the auricular vagus nerve by auricular acupuncture can improve the excitability of the motor cortex (Zhang et al., 2022). Based on this premise, the current study utilized interference current to stimulate both bilateral cervical vagus nerves, and innovatively found that tcVNS can increase the excitability of the motor cortex. This was demonstrated by the increased MEP amplitude and the shortened latency, indicating the activation of the corticospinal tract.

Many studies have used animal models to investigate the central nervous mechanism of VNS and considered that VNS activates the basal nucleus (Lyubashina and Panteleev, 2009), locus coeruleus (Hilz, 2022), and dorsal raphe nucleus (Hulsey et al., 2019) through the nucleus tractus solitarius (NTS) after the vagus nerve (Cooper et al., 2021). This causes an extensive release of norepinephrine, acetylcholine, serotonin, and other neurotransmitters in the cortex, combined with specific rehabilitation tasks targeted to promote synaptic plasticity in these neural pathways (Hulsey et al., 2019). Some researchers have found that inhibition of norepinephrine re-uptake could enhance excitability of the corticospinal tract, which may be the physiological mechanism whereby VNS promotes cortical excitability (Roosevelt et al., 2006; Kuo et al., 2017).

However, few studies have investigated the direct effect of nVNS on brain excitability. MEP is a reliable indicator to measure cortical excitability (Tang et al., 2019; Zhang J. et al., 2022; Zhang M. F. et al., 2022). We used latency and amplitude to evaluate the effect of VNS on the excitability of the motor cortex and used sham stimulation to eliminate possible placebo effects. The MEP amplitude of the real stimulus condition was significantly higher than that before the tcVNS. No significant changes was found on sham stimulation condition. MEP amplitude may be affected by both long term inhibitory and excitatory effects induced by TMS stimulation (Di Lazzaro et al., 2004; Mercante et al., 2015). Some previous studies reported that VNS induced short interval intracortical inhibition (Capone et al., 2015; van Midden et al., 2023). So that the MEP amplitude cannot sensitively reflect the changes of M1 plasticity (Vallence et al., 2023). The change of MEP latency can compensate for the lack of MEP amplitude. Latency reflects the time required for intra-cortical processing, corticocortical conduction, spinal processing, and neuromuscular transmission, depending on the state of the corticospinal system at the time of TMS delivery. The change of state at any point along the corticospinal pathway may affect the MEP latency (Hirano et al., 2016).

We used a crossover study design and a random stimulus sequence to ensure that different participants and the stimulus sequence did not affect the experimental results. The interval between the two stimulus conditions should be at least 24 h to avoid possible legacy effects. Considering the parasympathetic effect of the vagus nerve, the safety of stimulating bilateral vagus nerve simultaneously is still controversial. In previous studies, unilateral VNS has mostly been selected. One previous study reported that bilateral nVNS through the neck is safe (Nagami et al., 2019). In addition, previous reports have used a pulse current to stimulate the cervical vagus nerve (Dawson et al., 2021; De Ridder et al., 2021). However, we used a 50-Hz interference current, which is more comfortable than the pulse current interference wave because it does not cause muscle contraction.

This study also has several limitations. First, we did not attempt to investigate the optimal parameters for tcVNS treatment, such as intensive, duration. Second, only the MEP of the motor cortex of the left hemisphere was measured while both vagus nerves were stimulated bilaterally. Third, the sample size of this study was small, and the participants were mostly 20–30 years old, fewer male subjects. Fourth, this study only carried out a single stimulation, and the long-term effect of VNS on the motor cortex remains unclear. In addition, most participants were able to distinguish between real and sham stimulation due to the significant differences in intensity. Therefore, a single-blind design was employed in this study. Finally, this study suggested the activation of tcVNS on the corticospinal tract, but the specific neural pathway remains unknown. We need to explore this point in a follow-up study further.

## 5. Conclusion

In conclusion, bilateral tcVNS could safely and effectively increase the excitability of the motor cortex and may become a new treatment to improve motor function in central neural injured patients.

## Data availability statement

The original contributions presented in the study are included in the article/supplementary material, further inquiries can be directed to the corresponding author.

## Ethics statement

The studies involving humans were approved by the Ethics Committee of Yuedong Hospital, the Third Affiliated Hospital of Sun Yat-Sen University. The studies were conducted in accordance with the local legislation and institutional requirements. The participants provided their written informed consent to participate in this study.

## Author contributions

Z-MT is the guarantor of this study. YO and Z-MT participated in the conception and design of the study and also participated in the critical revision of the manuscript. M-XW was stimulation operator and AW was evaluator. Y-WZ contributed to participant recruitment. X-SG and ZH were involved with data collection. M-FZ and Z-YP finished data analysis. M-XW and AW prepared the first draft of this manuscript. All authors read and approved the final manuscript.
